
JOURNAL INFORMATION
==============================
NLM Title Abbreviation: ScientificWorldJournal
Journal ID: ScientificWorldJournal
NLM Journal ID: 101131163
Journal ID: TSWJ

The Scientific World Journal

ISSN: 2356-6140
EISSN: 1537-744X
Publisher: Wiley

ARTICLE INFORMATION
==============================
PMCID: PMC6084036
PMID: 30147512
DOI: 10.1100/tsw.2001.183
Article ID: 314903
Article version: 1
Subjects: Short Report

Muscular Abnormalities in the HIV-1 Transgenic Rat

Denaro F.J. *
Hayes N.
Jones O.
McCready M.
Gallo R.
Davis H.
Bryant J.
Institute of Human Virology, Baltimore, MD, USA
*F.J. Denaro: denaro@umbi.umd.edu
Publication date: 2001
Electronic publication date: 2001 Nov 13
Volume: 1
First page: 51
Last page: 51
Copyright: Copyright © 2001 F.J. Denaro et al.
Copyright year: 2001
License: This is an open access article distributed under the Creative Commons Attribution License, which permits unrestricted use, distribution, and reproduction in any medium, provided the original work is properly cited.
License URL: https://creativecommons.org/licenses/by/3.0/

Funding: National Institutes of Health NS31857 National Institutes of Health MH29494

==============================
Miami *Nature Biotechnology* Short Reports
*TheScientificWorld* (2001) 1 (S3), 51SR
ISSN 1532-2246; DOI 10.1100/tsw.2001.183

### MUSCULAR ABNORMALITIES IN THE HIV-1 TRANSGENIC RAT

F.J. Denaro*, N. Hayes, O. Jones, M. McCready, R. Gallo, H. Davis, and J. Bryant

Institute of Human Virology, Baltimore, MD
* Denaro@umbi.umd.edu

**INTRODUCTION.** Special attention is focused on the central nervous system (CNS) in AIDS because of the debilitating symptoms and unique neuropathology. Anti- viral therapy has been effective in treating many of the symptoms of AIDS. But the CNS and PNS can continue to display debilitating symptoms even when the patient is on antiviral therapy (1). Motor abnormalities and atrophy of skeletal muscle are two PNS problems that continue to resist treatment. In order to understand the disease process and to develop new therapies, animal model systems are needed (2). The recently developed HIV-1 Transgenic Rat (HIV-1 TgR) displays many neurological symptoms of AIDS (3). These symptoms include muscular wasting, weakness, difficulties in gait, and paralysis. Examination of the peripheral nerve has revealed degeneration of the axons and focal demyelination. The muscular changes may be secondary to this nerve pathology or myopathic mechanisms may be operational. The muscle in these animals was examined by histologic means. By identifying the type of degeneration it may be possible to eventual study the effect of growth factors or therapeutics.

**METHODS.** HIV-1TGRs were constructed using transgenic protocols. The pronuclei were microinjected with an HIV-1-gag-pol-pEVd 1443 clone in which the SphI and Bal1 fragment that contained the 3’ gag region and 5’ pol region of the infectious proviral DNA pNL4.3 clone was removed. This produced a non-infectious model. Gp-120 is found in their serum. Immunocytochemistry to Gp-120, TAT and Nef localized the viral products to the lymphoid tissue. Animals were observed for neurologic abnormalities. Their clinical symptoms were recorded over time. Both nerve and muscle were removed at necropsy for histologic analysis. Tissues were prepared for light and electron microscopy. The special stains used were H&E, NADH, ATPase, lymphocyte markers, and ApopTag. Thick sections stained with Toluidine Blue.

**RESULTS.** Depending on the muscle studied one could observe mild changes (inflammatory changes, and apoptosis to endothelial cells) to severe end stage changes (fatty replacement of muscle, phagocytosis of myocytes and necrosis). The hind limb muscle typically displayed the most marked changes. Intermediate changes include atrophic, angulated fibers (Fig.1), hypertropic fibers surrounded fat, increased connective tissue and internal nuclei (Fig. 2).

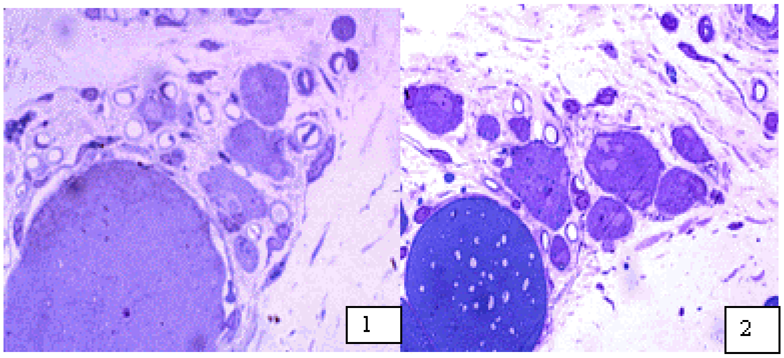

**DISCUSSION.** Patients with AIDS can present with motor problems and skeletal muscle wasting. Examination of the muscle in this new model demonstrates a group of symptoms similar to what has been reported for patients with AIDS. The HIV1-TGR is a small non-infectious model in which neurological and behavioral problems can be studied. Because of this it can satisfy many emerging research requirements that other models cannot address.

**ACKNOWLEDGEMENTS.** Supported by IHV and NIH grant NS31857 and MH29494.

**REFERENCES.**

1. Enzensberger, W. (1999) Eur. J. Med. Res. 11, 456-462
2. Vitkovic, L. et al. AIDS Res. Hum. Retroviruses 2(6), 753
3. Reid, W. et al. (2000) J. Hum. Virol. 3(5), 254
